# Fatal intoxication caused by the application of the multiple transdermals patchs of fentanyl

**DOI:** 10.11604/pamj.2015.20.21.5886

**Published:** 2015-01-07

**Authors:** Issam Serghini, Youssef Qamouss, Mohamed Zoubir, Jaafar Salim Lalaoui, Mohamed Boughalem

**Affiliations:** 1 Pôle Anesthésie-Réanimation, Hôpital Militaire Avicenne, Faculté de Médecine et de Pharmacie, Université Cadi Ayyad, 40010 Marrakech, Maroc

**Keywords:** Fentanyl, transdermal patch, respiratory insufficiency, fatal intoxication

## Abstract

Fentanyl (N-phenyl-N-(1-2-phenylethyl-4-piperidyl)propanamide) is a potent synthetic narcotic analgesic. He has an analgesic effect 100 times greater than that of morphine. The use of transdermal fentanyl delivrery systems has increased over recent years especially in patients with chronic pain who are already treated with high doses of morphine or it is derivate. However, many cases of fentanyl intoxication through a variety of transderrmal systems have been reported. This paper reports a fatality due to excessive administered Fentanyl Sandoz^®^ Matrix 50µg/h transdermal therapeutic systems.

## Introduction

Fentanyl is a potent opioid narcotic which was first synthesised by Janssen in 1959. The drug is a µ-opioid receptor agonist and is estimed to be 80 times more potent than morphine as analgesic. Fentanyl in a rate controlling membrane (RCM) transdermal patch form has been availble since the early 1990s for outpatient management of chronic pain. Fatalities associated with misuse or overuse of fentanyl patches have been reported [1].

### Patient and observation

**Figure 1:**
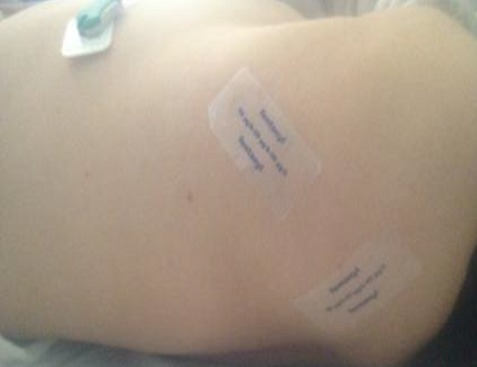
2 patch attached to her back

**Figure 2:**
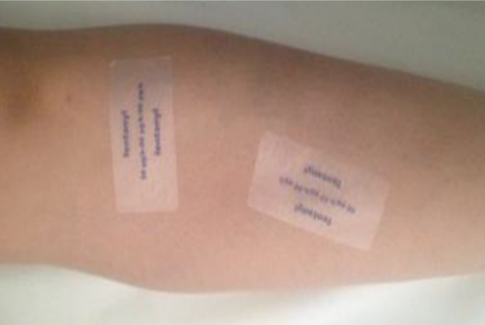
2 patch attached to her left leg

We report the case of a 73-year- old Moroccan women weighing 81.8 kg, was admitted to our Emergency departement exhibiting a comatose state, that have developed 6 hours previously. He had a history of asthma and rheumatic pain. His medications were Salbutamol by inhalation as needed for asthma and nonsteroidal antiinflammatory drugs as needed for the rheumatic pain. On examination, the first neurologic investigation confirmed a GCS-Score of 3, her pupils were pinpoint and the head atraumatic. The respiratory rate 10 breaths per minute, and the oxygen saturation 85% while he was breathing 4 liters of oxygen by nasal. The blood pressure was 80/60mm Hg, the heart rate was 42 beats/mn. The temperature was 37.3°C. During the initial resuscitation, she was intubated without difficulty using rapid sequence intubation and she was placed on mechanical ventilation. The Biochemistry results demonstrated a metabolic acidosis (pH 7.18; HCO3: 12 mmol/l) with hyperlactatemia (13 mmol/l) and acute renal failure (creatinine: 481 µmol/l), and a high potassium level of 5,9 mmol/l. The lumbar puncture was normal. The Computed tomography scan of the brain was normal. Upon detailed physical examination, we found 8 fentanyl transdermal thérapeutic systems (fentanyl Sandoz 50µg/h): 2 patch attached to her back (Figure 1) and 2 at her left leg (Figure 2). Summing up the symptoms of respiratory insufficiency, bradycardia, hypotension, unconsciousness, and miosis, we diagnosed opioid intoxication by a fentanyl patch. The resuscitation was immediately initiated. Because the patient was already intubated, we did not administer naloxone as antagonist concerning the short duration of action. During the next 6 hours, the blood pressure was stabilized with high doses of epinephrine 5 mg/h to reach an MAP of =75mmHg, but the patient was anuric with persistant renal failure (plasma creatinin 515 µmol/l). Decision was made with the nephrologist to start a continuous veno-venous hemodialysis (CVVH). The evolution was good. The patient was weaned from the mecanical ventilator and extubated after 4 days. She reported that her friend, who had colon cancer give her a few patches to decrease her chronic rheumatic pain. The patient discharged from the hospital after 7 days without sequelae.

## Discussion

Fentanyl is a pure and selective opioid µ receptor agonist that is 80-100 times more potent than morphine [2]. Fentanyl is administered via intravenous, epidural, transmucosal, and transdermal routes. Fentanyl Sandoz^®^ Matrix is a transdermal system that provides continuous systemic delivery of fentanyl, a potent opoid analgesic, for up to 72 hours. The patche are available as 2.1, 4.2, 6.3, 8.4, 12.6 and 16.8 mg patches that deliver 12, 25, 37, 50, 75 and 100µg/h, respectively. Since its introduction, transdermal fentanyl has been widely used for the management of chronic pain in patients that require opiate analgesia that cannot be managed adequately with less intensive analgesic therapy [3]. However,many cases of fentanyl intoxication through a variety of transdermal systems have been reported. Abusers have shown a variety of methods to violate the transdemal system, thus delivering fentanyl in an uncontrolled manner, sometimes yielding a fatal outcome [4]. Marquardt et al reported a case of fatal fentanyl poisoning in which the drug was scraped from the patch, heated, then inhaled [5]. DeSio reported a case of intravenous abuse of fentanyl after aspiration of the drug from patch [6]. Edinboro reported a fatal fentanyl intoxication caused by the application of multiple transdermal patches [7]. Kramer reported a fatality in a patient with respiratory arrest who was found to have a patch in the buccal cavity and a secon patch on the thigh [8]. Parucker reported a case of intentional oral ingestion of the contents of the patch that resulted in respiratory arrest. The patient had apparently bitten a patch [9]. Flannagan reported a case of fatal fentanyl poisoning where the decedent, a funeral home employe, apparently obtained the drug from a fentanyl patch that had been on deceased patient [10].

These case reports show the transdermal fentanyl system can be abused [4]. Respiratory depression, and to a lesser degree, circulatory depression are chief hazards of opiate agonist therapy. Opiate agonist overdose usually produces central nervous system depression that ranges from stupor to profond coma; respiratory depression; cold, clammy skin and/or hypothermia; bradycardia and hypotension [4]. In particular, a patient with asthma or another pulmonary disease may be at risk of respiratory depression at a lower fentanyl concentration. In general, respiratory depression occurs in opioid-naïve patients after acute fentanyl administration, in contrast to chronic management of pain, which has no significant risk of respiratory depression due to the patient's tolerance for the drug [11]. In our case report, the patient was naive opiate and had a history of pulmonary disease. Treatment of opiate agonist overdose includes reestablishment of adequate respiratory exchange by maintaining an adequate patent airway.

Other immediate supportive ans symptomatic treatment should be initiated based on the presentation. Respiratory depression may be traited with parenteral naloxone hydrochloride [4]. Naloxone, a pure opioid antagonist, can effectively reverse respiratory depression and therefore preventdeath. In this case, because the patient was already intubated, we did not administer naloxone as antidote concerning the short duration of action. Additionally, response to naloxone is helpful for making the diagnosis and thereby preventing an unnecessary examination [12]. Therefore, removal of the patch does not result in complete elimination of exposure to fentanyl, and the adverse effects, especially respiratory depression, can re-occur. Therefore, physicians must closely observe patients with significant symptoms of opioid intoxication due to transdermal fentanyl, such as respiratory depression or central nervous system depression, for at least 24 h after removal of the patch [12].

## Conclusion

The case history and toxicological findings of a fatal fentanyl intoxication due to the application of multiple transdermal patches are presented. This case demonstrates the need for caution in self-administration of transdermal fentanyl patches, in particular, the dangers inherent in the application of multiple patches which can result in the release of potentially toxic or lethal doses.

## References

[1] Coopman V, Cordonnier J, Pien K, Varenberghb DV (2007). LC MS/MS analysis of fentanyl and norfentanyl in a fatality due to application of multiple Durogesic^®^ transdermal therapeutic systems. Forensic Science International..

[2] Bullingham RES (1983). Opiate analgesia.

[3] Roy SD, Flynn GL (1989). Transdermal delivery of narcotic analgesics: comparative permeabilities of narcotic analgesics through human cadaver skin. Pharm Res..

[4] Mary Lynn Arvanitis (2002). Transdermal fentanyl abuse and misuse. American Journal of Emergency Medicine.

[5] Marquaardt KA, Tharratt RS (1994). Inhalation abuse of fentanyl patch. J Toxicol Clin Toxicol..

[6] DeSio JM, Bacon DR, Peer G, Lema MJ (1993). Intravenous abuse of transdermal fentanyl therapy in a chronic pain patient. Anesthesiology.

[7] Edinboro LE, Poklis A, Trautman D, Lowry S, Backer R, Harvey CM (1997). Fatal fentanyl intoxication following excessive transdermal application. J Forensic Sci..

[8] Kramer C, Tawney M (1998). A fatal overdose of transdermally administered fentanyl. J Am Osteopath Assoc..

[9] Purucker M, Swann W (2000). Potential for duragesic patch abuse. Ann Emerg Med..

[10] Marquardt KA, Tharratt RS, Musallam NA (1995). Fentanyl remaining in a transdermal system following three days of continuous use. Ann Pharmacother..

[11] Inturrisi CE (2002). Clinical pharmacology of opioids fot pain. Clin J Pain..

[12] Moon JM, Chun BJ (2011). Fentanyl Intoxication Caused by Abuse of Transdermal Fentanyl. Journal of Emergency Medicine..

